# Lymphoepithelioma-Like Carcinoma of the Bladder: A Case Report and Review of the Literature

**DOI:** 10.1155/2013/356576

**Published:** 2013-04-23

**Authors:** Kenichi Mori, Tadasuke Ando, Takeo Nomura, Fuminori Sato, Hiromitsu Mimata

**Affiliations:** Department of Urology, Faculty of Medicine, Oita University, Idaigaoka 1-1, Hasama-cho, Yufu, Oita Prefecture 879-5593, Japan

## Abstract

Lymphoepithelioma-like carcinoma (LELC) in the bladder is uncommon with a reported incidence of 0.4%–1.3% of all bladder carcinomas. In Japan, some occurrences of LELC have been reported in the renal pelvis and ureter but only two in the bladder. A bladder tumor was identified in a 70-year-old man suffering from macroscopic hematuria for 2 months. Sections of the transurethral tumor resection showed invasive high-grade urothelial carcinoma. The patient was diagnosed with local invasive bladder tumor, and cystectomy with ileal conduit formation was performed. The final pathological evaluation was predominant LELC with urothelial carcinoma. We present a new case of LELC in the bladder and performed a review of all published cases of LELC in the urinary tract to obtain its characteristics and prognostic guide.

## 1. Introduction

Lymphoepithelioma is a form of undifferentiated nasopharyngeal carcinoma primarily described in Asian patients. In most cases, the carcinoma has a close pathogenetic link to Epstein-Barr virus (EBV). Tumors with similar histological features arising outside the nasopharynx are called lymphoepithelioma-like carcinoma (LELC). Although LELC occurs in various organs including the salivary glands, thymus, lung, skin, stomach, uterine cervix, and breast, its occurrence in the urinary system is very rare. LELC in the bladder is uncommon with a reported incidence of 0.4%–1.3% of all bladder carcinomas. In Japan, some occurrences of LELC have been reported in the renal pelvis and ureter [1–3] but only two in the bladder. These tumors are classified according to lymphoepithelioma component as pure (100%), predominant (≧50%), or focal (<50%) [4].

## 2. Case Presentation

**Figure 1:**
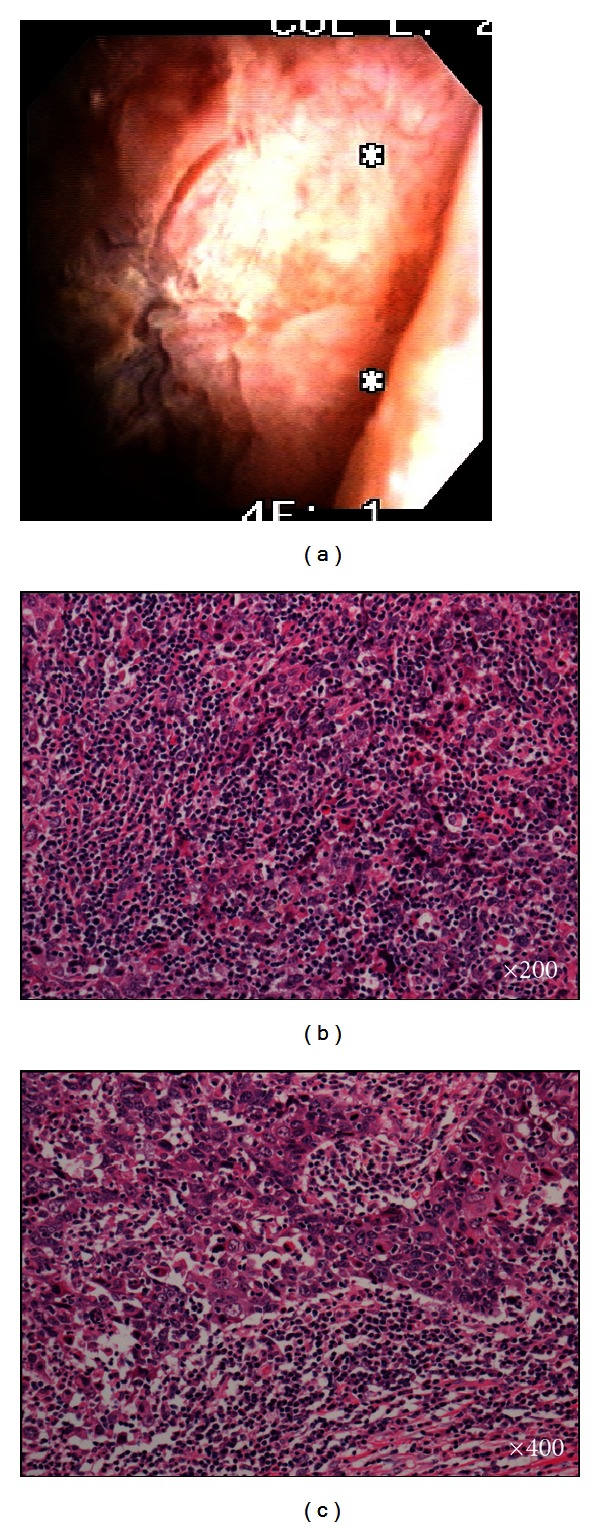
(a) Nonpolypoid tumor in the left lateral bladder wall. (b) Lymphoepithelioma-like carcinoma, syncytial pattern with prominent lymphocytic infiltrate. (c) Lymphoepithelioma-like carcinoma with urothelial carcinoma.

A 70-year-old man presented with macroscopic hematuria for 2 months. Urine cytology analysis showed markedly atypical urothelial cells. The patient underwent a flexible cystoscopic examination, which revealed a nonpolypoid tumor of 4 cm diameter in the left lateral bladder wall (Figure 1(a)). A computed tomography (CT) scan revealed nonlymph node metastasis, nondistant metastasis, and left hydronephrosis. This tumor was also examined by magnetic resonance imaging (MRI), which evidently showed bladder muscle invasion. Sections of the transurethral resection (TUR) showed invasive carcinoma, that is, grade 3 urothelial carcinoma. The patient was diagnosed with local invasive bladder tumor, and cystectomy with ileal conduit formation was performed. The final pathological evaluation of the tumor was predominant LELC (Figure 1(b)) with urothelial carcinoma (Figure 1(c)). EBV was not present in this case, and the patient did not receive adjuvant therapy. The patient is under close observation with regular clinical and radiologic followup and remains well after 10 months with no evidence of disease recurrence.

## 3. Literature Review

The total 103 cases comprised 86 of bladder (83.5%), 7 of pelvis (6.8%), 5 of prostate (4.9%), 4 of ureter (3.9%), and 1 of urethra (0.9%). The total 103 cases with LELC of urinary tract were reported between 1991 and 2012 [1–9]. Seventy-three males (70.9%) and 30 females (29.1%) with mean 68.9 years ranged from 44 to 90 years were included. 

The most common presenting symptom of LELC in the bladder is symptomatic hematuria, generally accompanied by urgency, and that of prostate is obstructive symptoms and elevated prostate-specific antigen (range, 4.2–19.4 ng/mL). 

Regarding the histological type, the pure type was 41/103 cases (39.8%), the mixed type with predominant and focal type was 47/103 cases (45.6%), and the others were not shown. In mixed type of the bladder, pelvis, ureter, and urethra, the most commonly coexisted histological subtypes were high-grade urothelial carcinoma, adenocarcinoma, and squamous carcinoma. In addition to LELC, the coexistent Gleason 3+3 (*n* = 1), 3+4 (*n* = 1), and 4+3 (*n* = 3) conventional acinar adenocarcinoma had a ductal component (*n* = 3) in the prostate. In situ hybridization was negative for EBV-encoded RNA in the 35 cases of all.

The patients with LELC were mostly diagnosed at high stages. Tumor stages of the bladder, pelvis, and urethra were as follows: 14/98 cases T1 (14.3%); 49/98 cases T2 (50.0%); 30/98 cases T3 (30.6%); 2/98 cases T4 (2.0%); the others were not shown. Regarding LELC of the prostate, 1/5 case was T1 (20%), 3/5 cases were T3 (60%), and 1/5 case was T4 (20%). All 85/103 cases (82.5%) were diagnosed at more than T2 stages. 

Treatment for LELC with bladder, pelvis, ureter, and urethra consisted of radical cystectomy in 34/98 cases (34.7%), partial cystectomy in 7/98 cases (7.1%), TUR in 46/98 cases (46.9%), nephroureterectomy in 6/98 cases (6.1%), nephrectomy in 2/98 (2.0%), ureterectomy in 1/98 (1%), and the other cases were unknown. Adjuvant therapies were chemotherapy in 28/98 (28.6%), radiotherapy in 17/98 (17.3%), and intravesical chemotherapy in 3/98 (3.1%). On the other hand, treatment for LELC with prostate consisted of radical prostatectomy in 2/5 cases (40%) and TUR in 3/5 cases (60%). In some case, LELC was unexpected finding at the time of TUR for benign prostatic hyperplasia. 

The mean followup was 21.7 months (range, 0–216 months) in 92 cases with LELC of bladder, pelvis, ureter, and urethra. Regarding the outcome, the 61/92 cases (66.3%) had no evidence of disease, 14/92 cases (15.2%) were tumor death, 9/92 cases (9.8%) had metastasis (lung, lymph nodes, skin, and abdomen), and 8/92 cases (8.7%) were death of other cause. In 4 cases with LELC of prostate, the mean duration was 20.3 months (range, 8–26 months). The outcomes of 4 cases were tumor death. 

## 4. Discussion

LELC often manifests in T2-T3 stages and occurs predominantly in male patients with an average age of 60 years. Although rates of metastases range from 10% to 15%, the present tumor was associated with a favorable prognosis and a 5-year survival of 59%, achieving 62% in the pure type [8]. The pure or predominant type responds better to chemotherapy than conventional urothelial carcinoma [5], and this provides a potential to salvage bladder function. This advantage is lost when the urothelial elements predominate. Lopez-Beltran et al. [7] reported that 2 of 3 patients with pure type received transurethral resection of the bladder and adjuvant chemotherapy. The both showed no evidence of disease at 21 and 47 months. Tamas et al. [5] reported that 2 of 3 pure cases received chemotherapy, and the both showed no evidence of disease at 4 and 65 months. In most cases, the platinum-based chemotherapies were performed [9]. Furthermore, a recent study also reported that patients treated with cystectomy had a prognosis similar to conventional urothelial carcinoma. Our case underwent radical cystectomy and no evidence of disease 10 months after surgery. In case of metastasis, the platinum-based chemotherapies will be scheduled. However, as with other variants of bladder tumors, there are no clear guidelines for the treatment of LELC. It is strongly suggested that clear guideline for LELC is needed.
